# Quercetin attenuates acute alcohol-induced liver injury in mice by modulating lipid metabolism, oxidative stress, and inflammation

**DOI:** 10.3389/fphar.2026.1702639

**Published:** 2026-02-18

**Authors:** Wenjing He, Binbin Zhang, Siwei Li, Yuanyuan Qian

**Affiliations:** 1 Department of Medical Intensive Care Unit, The Affiliated Hospital of Changchun University of Chinese Medicine, Changchun, China; 2 Department of Emergency Medicine, The Affiliated Hospital of Changchun University of Chinese Medicine, Changchun, China; 3 Department of Research and Development, Jilin Ruiguo Technology Co., Ltd., Changchun, China

**Keywords:** acute ethanol injury, alcoholic liver disease (ALD), inflammation, lipid metabolism, oxidative stress, quercetin

## Abstract

**Background:**

Alcoholic liver disease (ALD) remains a major global health burden, and effective therapeutic options are limited. Quercetin, a dietary flavonoid with antioxidant and anti-inflammatory properties, has shown hepatoprotective potential, but its mechanisms in acute alcohol-induced liver injury require clarification.

**Methods:**

Fifty male BALB/c mice were randomly divided into five groups (n = 10/group): control (CG), model (MG), low-dose quercetin (LQ, 50 mg/kg), high-dose quercetin (HQ, 100 mg/kg), and oyster soybean carnitine mice in the oyster soybean carnitine group (OG) were administered oyster soybean carnitine at a volume of 20 mL/kg.(OG, 20 mL/kg). All treatments were administered orally once daily for 14 days. Acute alcohol injury was induced on Day 14 using 52% (v/v) ethanol. Behavioral intoxication indices, serum biochemistry, liver oxidative stress markers, inflammatory cytokines, histopathology, lipid accumulation, and the expression of lipid-metabolismrelated genes and proteins (FAS, SCD1, ACC1, CPT1, PGC-1α, PPARα) were assessed.

**Results:**

HQ significantly prolonged alcohol tolerance time (P < 0.01) and shortened sober-up time (P < 0.01). HQ markedly reduced serum AST, ALT, TG, FFA, and glycerol levels (P < 0.01). Quercetin also lowered hepatic TG, TNF-α, and IL-6 and increased SOD, GSH, and CAT activities. Histological and Oil Red O staining confirmed reduced steatosis. Mechanistically, quercetin downregulated hepatic FAS, SCD1, and ACC1 while upregulating CPT1, PGC-1α, and PPARα at both mRNA and protein levels.

**Conclusion:**

Quercetin exerts hepatoprotective effects against acute alcohol-induced liver injury through coordinated regulation of lipid metabolism, oxidative stress, and inflammatory responses.The study did not include a quercetin-only group or measure blood ethanol levels, which should be addressed in future work.These findings support the potential of quercetin as a natural candidate for preventing alcohol-related hepatic injury.

## Introduction

Alcoholic liver disease (ALD) refers to a hepatic metabolic disorder induced by excessive alcohol consumption. In its initial phases, the condition commonly presents as hepatic steatosis, which may progress to more severe forms such as alcoholic hepatitis, fibrosis, and ultimately cirrhosis. Critical cases can result in acute alcohol intoxication and potentially fatal outcomes (Gao and Bataller, 2011; Louvet and Mathurin, 2015). Extensive research indicates that the pathogenesis of ALD involves multiple mechanisms, including oxidative stress, inflammatory reactions (Dong et al., 2020), disruption of intestinal homeostasis (Sharma et al., 2021), as well as aberrant lipid synthesis and ethanol metabolism (Teschke, 2018), all contributing to alcohol-induced liver damage. Notably, oxidative stress and inflammatory responses are recognized as central drivers of hepatotoxicity in ALD (Ye et al., 2021). Although chronic alcohol models better simulate long-term human ALD, the acute ethanol model is widely used to investigate early hepatocellular injury, oxidative stress, and metabolic responses. Acute models allow precise control of ethanol dose, rapid manifestation of hepatic damage, and clearer mechanistic interpretation without confounding chronic systemic alterations. Currently, clinical management strategies for ALD remain limited, and certain pharmacological treatments are associated with undesirable side effects. Hence, identifying safe and effective therapeutic agents for preventing and treating ALD holds substantial clinical importance.

Quercetin is widely found in various Chinese herbal medicines, as well as in fruits and vegetables. It is a typical flavonoid alcohol compound. It has antioxidant (Vidyashankar et al., 2013), anti-inflammatory (Chen, 2010), anti-thrombotic, anti-tumor (Angst et al., 2013) and other biological activities. It is considered effective in preventing diseases such as diabetes, cardiovascular disease, autoimmune diseases, and cancer (Dabeek and Marra, 2019; Guan et al., 2025). Numerous *in vivo* and *in vitro* experiments have demonstrated that quercetin can protect the nervous system, liver, and kidneys through its anti-oxidative stress and anti-inflammatory properties (Carrasco-Pozo et al., 2015; Shabbir et al., 2020; Taslidere et al., 2016; Zhang et al., 2014; Liu et al., 2020). Prior studies have demonstrated the protective effects of quercetin in non-alcoholic fatty liver disease (NAFLD/MASLD) and chronic ALD models. However, its integrated regulatory effects on lipid synthesis, β-oxidation, oxidative stress, and inflammatory responses in acute alcohol-induced liver injury remain inadequately explored.

Therefore, the present study aimed to systematically evaluate the hepatoprotective effects of quercetin in a mouse model of acute alcohol-induced liver injury. Behavioral intoxication assessments, serum biochemical analyses, hepatic oxidative stress and inflammatory markers, histopathology, and the expression of key lipid metabolism-related genes and proteins were examined to elucidate the underlying mechanisms. This study provides new mechanistic insights and supports the potential of quercetin as a natural candidate for preventing alcohol-related liver injury.

## Materials and methods

### Feeding and management of experimental animals

Fifty male BALB/c mice (18–22 g, 6–8 weeks old) were obtained from Liaoning Changsheng Biotechnology Co., Ltd. During the feeding period, they were fed freely at 21 °C–23 °C, and the light and dark cycle of the feeding environment was 12 h. After 1 week of acclimation, mice were randomly assigned to five groups (n = 10/group). All experimental procedures were approved by the Laboratory Animal Management and Use Committee of the Hubei Provincial Center for Disease Control and Prevention (Approval No.: 202410117).

### Main experimental reagents

Quercetin was obtained from Shaanxi Bolin Biotechnology Co., Ltd. 52% (v/v) commercial liquor was sourced from Niulanshan, Beijing Shunxin Agriculture Co., Ltd., while oyster soybean carnitine was acquired from Shenzhen Haiwang Health Technology Development Co., Ltd. Assay kits for AST/GOT, ALT/GPT, and glycerol were procured from Nanjing Jiancheng Bioengineering Institute. Triglyceride quantification kit and cDNA synthesis kit were supplied by Shanghai Biyuntian Biotechnology Co., Ltd. Additional reagents, including detection kits for free fatty acid (FFA), malondialdehyde (MDA), superoxide dismutase (SOD), reduced glutathione (GSH), catalase (CAT), as well as mouse TNF-α and IL-6 ELISA kits, along with an oil red O staining kit, were purchased from Beijing Solarbio Technology Co., Ltd. Hematoxylin and eosin staining solutions were provided by Wuhan Sevier Biotechnology Co., Ltd. Four percent paraformaldehyde and primer sequences were sourced from Biotechnology (Shanghai) Co., Ltd., and 2 × qPCR Mix (Green) was obtained from Heyuan Liji (Shanghai) Biotechnology Co., Ltd.

### Main experimental equipment

Electronic balance was purchased from Mettler-Toledo (Changzhou) Precision Instrument Co., Ltd.; microplate reader was purchased from Nanjing Detie Experimental Equipment Co., Ltd.; semi-automatic slicing machine was purchased from Jinhua Huasu Technology Co., Ltd.; the power supply of electrophoresis apparatus was purchased from Beijing Liuyi Biotechnology Co., Ltd.; the PCR instrument was purchased from Thermo, United States.

### Experimental animal grouping and sample collection

All mice received daily oral administration from Day 1 to Day 14. The low-dose quercetin group (LQ) and high-dose quercetin group (HQ) were treated with quercetin at 50 mg/kg and 100 mg/kg, respectively, doses within the range previously reported to exhibit protective effects in ethanol-induced liver injury models (Molina et al., 2003; Chen, 2010). The oyster soybean carnitine group (OG) received mice in the oyster soybean carnitine group (OG) were administered oyster soybean carnitine at a volume of 20 mL/kg.(20 mL/kg) oyster soybean carnitine, while the control group (CG) and model group (MG) were given 1% sodium carboxymethyl cellulose (CMC-Na) as vehicle. Although specific studies on oyster soybean carnitine are limited, compounds such as L-carnitine have been shown to ameliorate acute liver damage in rodent models (Demirdag et al., 2004). The detailed animal grouping and dosing schedules are summarized in Table 1.

**TABLE 1:** Animal grouping and dosing schedules.

Grouping	Group name	Mice intragastric administration of drugs	52% (v/v) ethanol by gavage (13.25 mL/kg/bw)
Blank control group	CG	1% sodium carboxymethyl cellulose	-
Model group	MG	1% sodium carboxymethyl cellulose	+
Low-dose quercetin group	LQ	Quercetin	+
High-dose quercetin group	HQ	Quercetin	+
Oyster soybean carnitine group	OG	Oyster soybean carnitine	+

On Day 14, 1 hour after the final administration, acute alcohol-induced liver injury was triggered by a single intragastric dose of 52% (v/v) ethanol at 13.25 mL/kg in the MG, LQ, HQ, and OG groups. This procedure is consistent with previously established acute ethanol gavage models in mice, in which single high-dose ethanol administration induces acute liver injury within hours. The CG received an equal volume of purified water instead of ethanol. Six hours after ethanol administration, all animals were euthanized for blood and tissue sample collection. Quercetin administration followed a pre-treatment regimen, aimed at evaluating its preventive effects against acute alcohol challenge.

### Drunk and sober observation

After ethanol administration, the time of giving alcohol, the time of disappearance of righting reflex (the mice kept their limbs up and their backs down for 30 s, which was drunk) and the recovery time of righting reflex (the mice could turn over after a period of time) were recorded. The alcohol tolerance time (righting reflex disappearance time-drinking time) and sober-up time (righting reflex recovery time-drinking time) were calculated.

### Blood biochemical analysis

Blood was collected from the retro-orbital sinus under isoflurane anesthesia, yielding approximately 0.8–1.0 mL per mouse. Samples were allowed to clot at room temperature for 30 min and centrifuged at 3,000 rpm for 15 min at 4 °C. Serum levels of AST, ALT, TG, FFA, and glycerol were measured according to the manufacturers’ instructions. All assays were performed in triplicate.

### Liver biochemical analysis

An appropriate amount of liver tissue was weighed and placed in an appropriate amount of extract for ice bath homogenization, and the supernatant was taken. The contents of TG, MDA, SOD, GSH, CAT, TNF-α, and IL-6 in liver tissue were determined according to the kit instructions.

### Histopathological analysis

Liver and kidney tissues were fixed in 4% paraformaldehyde for 24 h, dehydrated, embedded in paraffin, and cut into 4 μm sections. Samples were stained with hematoxylin for 5 min and eosin for 2 min.

### Liver fat analysis

The liver samples were processed following the manufacturer’s protocol provided with the Oil Red O staining kit. Subsequent microscopic examination was conducted using a light microscope to evaluate the staining outcomes.

### Detection of liver RT-qPCR

Total RNA was isolated employing the Trizol reagent, followed by reverse transcription into complementary DNA (cDNA) with a commercial reverse transcription kit. Subsequently, quantitative PCR was performed to measure the mRNA expression of genes associated with both fatty acid biosynthesis and β-oxidation pathways in hepatic tissues. All reactions were conducted in triplicate to ensure reproducibility. For normalization purposes, β-actin served as the internal control gene, and the relative expression levels of target genes were calculated using the 2^−ΔΔCT^ method. The specific primer sequences utilized for PCR amplification are listed in Table 2.

**TABLE 2:** Primers for PCR.

Gene	Forward primer sequence (5′–3′)	Reverse primer sequence (5′–3′)
FAS	TGT​CCT​GCC​TCT​GGT​GCT​TG	TTT​CAC​GAA​CCC​GCC​TCC​TC
SCD1	CGT​GGG​TTG​GCT​GCT​TGT​G	CGG​GCT​TGT​AGT​ACC​TCC​TCT​G
ACC1	TGG​AAG​TCG​GCT​ATG​GAA​ATT​GC	TTG​TCA​GGA​AGA​GGC​GGA​TGG
CPT1	ACG​GCA​GAG​CAG​AGG​TTC​AAG	ACA​CCA​CAT​AGA​GGC​AGA​AGA​GG
PGC-1*α*	AAG​ACA​CCT​TCC​TCT​CCT​CCT​TC	CAC​CAA​CCA​GAG​CAG​CAC​AC
PPAR*α*	CGA​TGC​TGT​CCT​CCT​TGA​TGA​AC	TCA​CAG​AAC​GGC​TTC​CTC​AGG
*β*-actin	ACT​GCC​GCA​TCC​TCT​TCC​TC	AAC​CGC​TCG​TTG​CCA​ATA​GTG

### Western blot analysis

Proteins were extracted from liver tissues using lysis buffer supplemented with protease inhibitors. Equal amounts of protein (20 μg) were separated by SDS–polyacrylamide gel electrophoresis (SDS-PAGE) and transferred onto polyvinylidene difluoride (PVDF) membranes. After blocking with 5% non-fat milk, the membranes were incubated overnight at 4 °C with primary antibodies against fatty acid synthase (FAS; 1:1000, Cell Signaling Technology, MA, United States), stearoyl-CoA desaturase-1 (SCD1; 1:1000, Abcam, United Kingdom), acetyl-CoA carboxylase 1 (ACC1; 1:1000, Cell Signaling Technology, MA, United States), carnitine palmitoyltransferase-1A (CPT1A; 1:2000, Abcam, UK), peroxisome proliferator-activated receptor-γ coactivator-1α (PGC-1α; 1:1000, Abcam, United Kingdom), peroxisome proliferator-activated receptor-α (PPARα; 1:1000, Abcam, United Kingdom), and β-actin (1:5000, Proteintech, Wuhan, China). After washing three times with washing buffer, the membranes were incubated with HRP-conjugated anti-rabbit IgG (1:5000, Cell Signaling Technology, MA, United States) or HRP-conjugated anti-mouse IgG (1:5000, Cell Signaling Technology, MA, United States) for 2 h at room temperature. Protein bands were visualized using an enhanced chemiluminescence (ECL) detection system and quantified using ImageJ software, with β-actin serving as the internal loading control.

### Statistical analysis

The results for each experimental group are presented as mean values accompanied by the standard deviation. Statistical analyses were conducted using GraphPad Prism software (version 8.0.1), with between-group comparisons performed via one-way ANOVA. A significance threshold of P < 0.05 was applied to determine statistically notable differences. (Note: an asterisk (*) denotes comparisons against the blank control group, while a hash symbol (#) indicates comparisons relative to the model group.)

## Results

### Effects of quercetin on alcohol tolerance time and sober-up time in acute drinking mice

**FIGURE 1:**
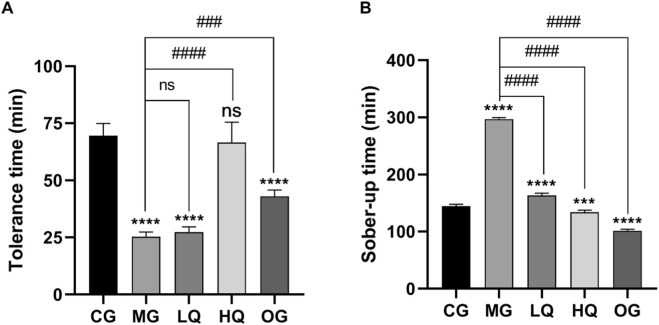
Effects of quercetin on alcohol tolerance time **(A)** and sober-up time **(B)** in mice subjected to acute alcohol exposure. Data are expressed as mean ± SD (n = 10). *P < 0.05 and *P < 0.01 vs. control group (CG); #P < 0.05 and ##P < 0.01 vs. model group (MG).

It can be seen from Figure 1 that following acute ethanol administration, mice in the model group (MG) exhibited a significantly shortened alcohol tolerance time and prolonged sober-up time compared with the control group (CG). Pretreatment with quercetin markedly improved behavioral intoxication parameters in a dose-dependent manner. In particular, high-dose quercetin (HQ) significantly prolonged the alcohol tolerance time and shortened the sober-up time compared with the MG group (P < 0.01). The effects observed in the HQ group were comparable to those of the oyster soybean carnitine group (OG), indicating a protective role of quercetin against acute alcohol-induced intoxication.

### Effect of quercetin on blood biochemical indexes in acute drinking mice

**FIGURE 2:**
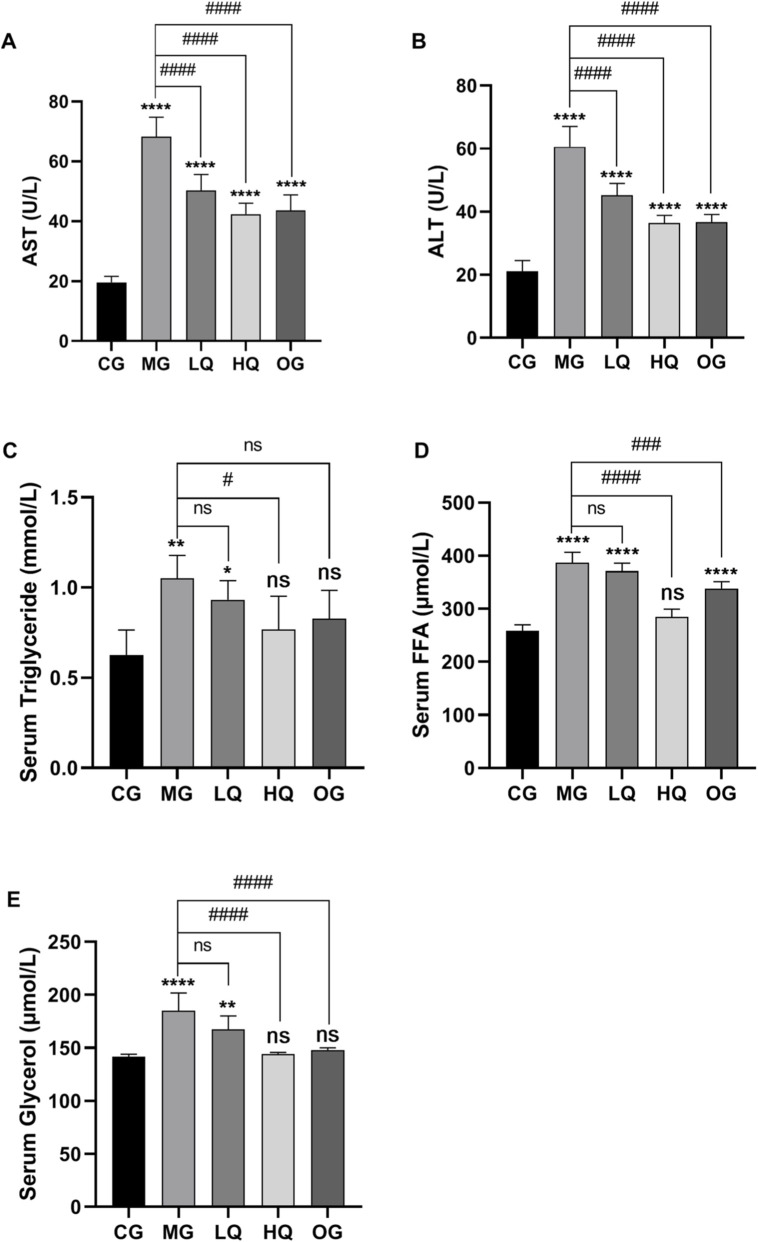
Effects of quercetin on serum biochemical parameters in mice with acute alcohol exposure. Serum levels of aspartate aminotransferase AST, **(A)**, alanine aminotransferase ALT, **(B)**, triglycerides TG, **(C)**, free fatty acids FFA, **(D)**, and glycerol **(E)** were determined. Data are expressed as mean ± SD (n = 10). *P < 0.05 and *P < 0.01 vs. CG; #P < 0.05 and ##P < 0.01 vs. MG.

As illustrated in Figure 2, serum levels of AST, ALT, triglyceride, free fatty acid, and glycerol were markedly elevated in the MG group relative to the CG group (P < 0.01). In the LQ group, a significant reduction in serum AST and ALT was observed compared to the MG group (P < 0.01), along with a downward trend in triglyceride, free fatty acid, and glycerol levels, though these changes did not reach statistical significance (P > 0.05). By contrast, the HQ group exhibited a substantial decrease in serum triglyceride content (P < 0.05), while AST, ALT, free fatty acid, and glycerol levels were all significantly reduced (P < 0.01). Similarly, the OG group demonstrated pronounced declines in serum concentrations of AST, ALT, free fatty acids, and glycerol (P < 0.01).

### Effects of quercetin on liver coefficient and liver function indices in acute drinking mice

**FIGURE 3:**
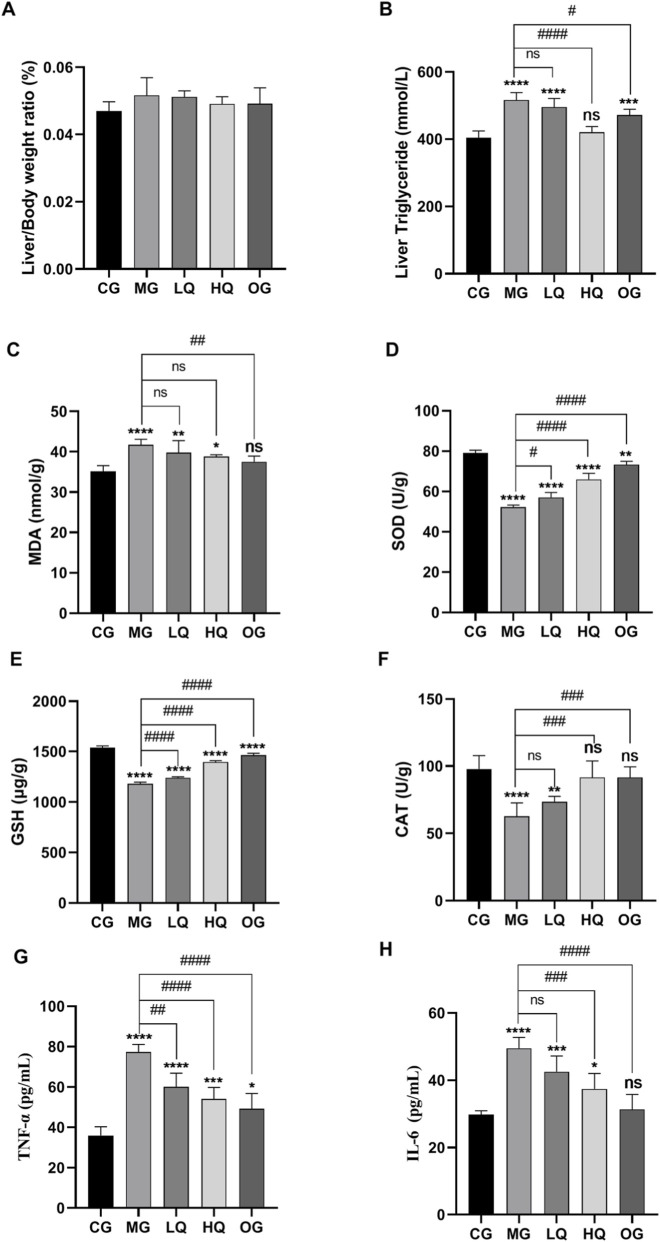
Effects of quercetin on liver coefficient and hepatic biochemical indices in mice with acute alcohol exposure. Liver coefficient **(A)**, hepatic triglyceride content **(B)**, malondialdehyde MDA, **(C)**, superoxide dismutase SOD, **(D)**, glutathione GSH, **(E)**, catalase CAT, **(F)**, tumor necrosis factor-α TNF-α, **(G)**, and interleukin-6 IL-6, **(H)** were measured. Data are expressed as mean ± SD (n = 10). *P < 0.05 and *P < 0.01 vs. CG; #P < 0.05 and ##P < 0.01 vs. MG.

As illustrated in Figure 3, the liver index in the MG group was elevated relative to the CG group, although the difference did not reach statistical significance (P > 0.05). In comparison with the MG group, reductions in liver coefficient were observed in the LQ, HQ, and OG groups; however, these changes were not statistically significant (P > 0.05). Hepatic triglyceride levels were markedly higher in the MG group than in the CG group (P < 0.01). Relative to the MG group, triglyceride content in the LQ group did not decrease significantly (P > 0.05), whereas a highly significant reduction was noted in the HQ group (P < 0.01), and a significant decrease was observed in the OG group (P < 0.05). With respect to oxidative stress markers, MDA levels were significantly elevated in the MG group compared to the CG group (P < 0.01), while activities of the antioxidant enzymes SOD, GSH, and CAT were considerably suppressed (P < 0.01). Compared to the MG group, MDA content declined in both the LQ and HQ groups without statistical significance (P > 0.05), but a pronounced reduction was detected in the OG group (P < 0.01). SOD activity rose significantly in the LQ group (P < 0.05), and extremely significantly in the HQ and OG groups (P < 0.01). GSH levels were markedly elevated across all treatment groups (LQ, HQ, OG; P < 0.01). CAT activity also showed significant enhancement in the HQ and OG groups (P < 0.01). Regarding inflammatory cytokines, concentrations of TNF-α and IL-6 were substantially higher in the MG group than in the CG group (P < 0.01). In contrast, TNF-α levels were significantly lower in the LQ, HQ, and OG groups relative to the MG group (P < 0.01). Similarly, IL-6 levels were markedly reduced in the HQ and OG groups (P < 0.01).

### Effects of quercetin on pathological sections of liver and kidney in acute drinking mice

**FIGURE 4:**
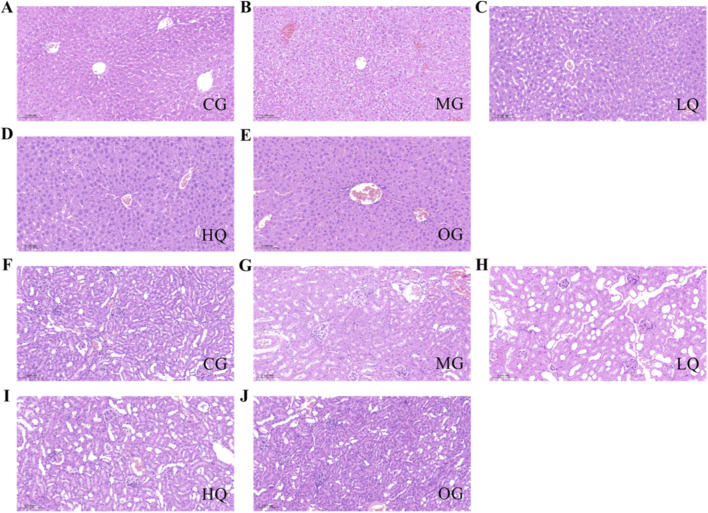
Effects of quercetin on histopathological changes in liver and kidney tissues of mice with acute alcohol exposure. Representative hematoxylin and eosin (H&E)-stained sections of liver **(A–E)** and kidney **(F–J)** tissues are shown (original magnification, ×200).

As illustrated in Figure 4, hepatic lobule architecture in the CG group exhibited well-defined organization, with hepatocytes arranged in an orderly manner along the hepatic plates. These cells displayed uniform morphology and structural integrity, radiating peripherally from the central vein alongside hepatic sinusoids. Nuclei were prominent, rounded, and centrally positioned, with no signs of steatosis. In contrast, the MG group showed disorganized hepatic lobules, irregular hepatic plate arrangement, and variably sized lipid droplets within the cytoplasm. Nuclei were displaced to the periphery, accompanied by prominent fat vacuolation and ballooning degeneration of hepatocytes. Hepatocytes in the LQ group demonstrated comparatively preserved morphological structure relative to the MG group, though minor fat vacuolation and ballooning changes were still observed. The HQ group exhibited near-normal cellular architecture, with distinct nuclei, neatly arranged hepatic cords, and minimal lipid accumulation. Similarly, the OG group displayed essentially restored hepatocyte morphology, clear nuclei, organized cord-like structures, narrowed sinusoids, and only occasional fat vacuoles. Renal histology in the CG group revealed regularly aligned tubular epithelial cells, normal Bowman’s space, and uniform renal corpuscles. The MG group, however, exhibited widespread swelling, dilatation, and marked deformation of renal tubules, accompanied by varying degrees of luminal narrowing. Compared to the MG group, renal cellular morphology in the LQ group was largely maintained, with limited tubular epithelial swelling and vacuolar degeneration. Both the HQ and OG groups showed essentially normal renal structure, with only slight swelling in a minimal number of tubular epithelial cells.

### Effect of quercetin on liver fat in acute drinking mice

**FIGURE 5:**
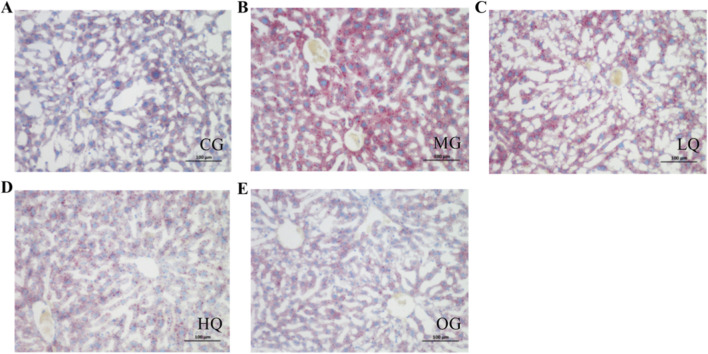
Effects of quercetin on hepatic lipid accumulation in mice with acute alcohol exposure. Representative images of hepatic lipid deposition are shown **(A-E)** (original magnification, ×200).

As illustrated in Figure 5, hepatocytes in the CG group exhibited blue staining in both the cytoplasm and nucleus, with minimal presence of red lipid droplets. In contrast, the MG group displayed numerous diffusely distributed red lipids of varying sizes within the cytoplasm. The LQ group showed a reduction in both the amount and staining intensity of cytoplasmic lipid droplets. Hepatocytes in the HQ and OG groups contained only scant, finely dispersed red lipids, appearing most similar to those in the CG group.

### Effect of quercetin on fatty acid synthesis in liver of acute drinking mice

**FIGURE 6:**
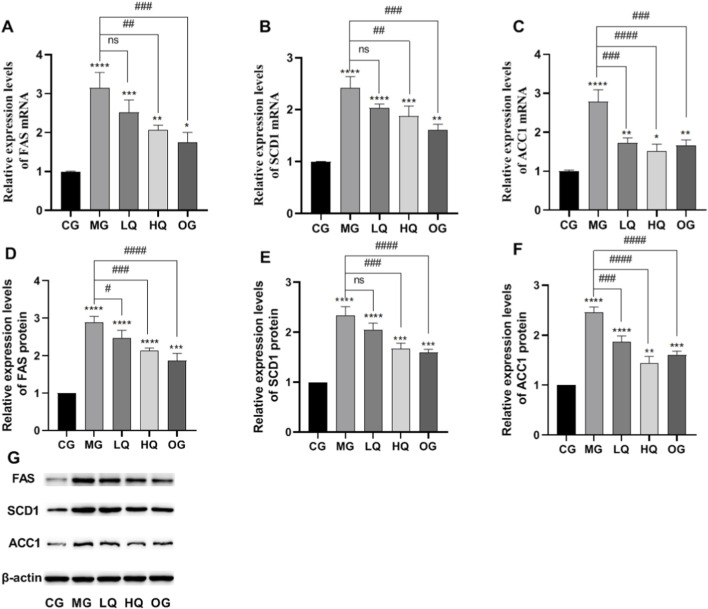
Effects of quercetin on hepatic fatty acid synthesis in mice with acute alcohol exposure. The mRNA expression levels of fatty acid synthase FAS, **(A)**, stearoyl-CoA desaturase-1 SCD1, **(B)**, and acetyl-CoA carboxylase-1 ACC1, **(C)** were determined by RT-qPCR. Protein expression levels of FAS **(D)**, SCD1 **(E)**, and ACC1 **(F)** were analyzed by Western blotting, and the corresponding densitometric quantification is shown **(G)**. Data are expressed as mean ± SD (n = 10). *P < 0.05 and *P < 0.01 vs. CG; #P < 0.05 and ##P < 0.01 vs. MG.

As illustrated in Figure 6, hepatic mRNA and protein expression levels of FAS, SCD1, and ACC1 were markedly elevated in the MG group compared to the CG group (P < 0.01). In contrast, administration of quercetin (HQ group) and the positive control (OG group) significantly reduced the expression of these lipid metabolism-related genes and proteins relative to the MG group (P < 0.01).

### Effect of quercetin on fatty acid β-oxidation in liver of acute drinking mice

**FIGURE 7:**
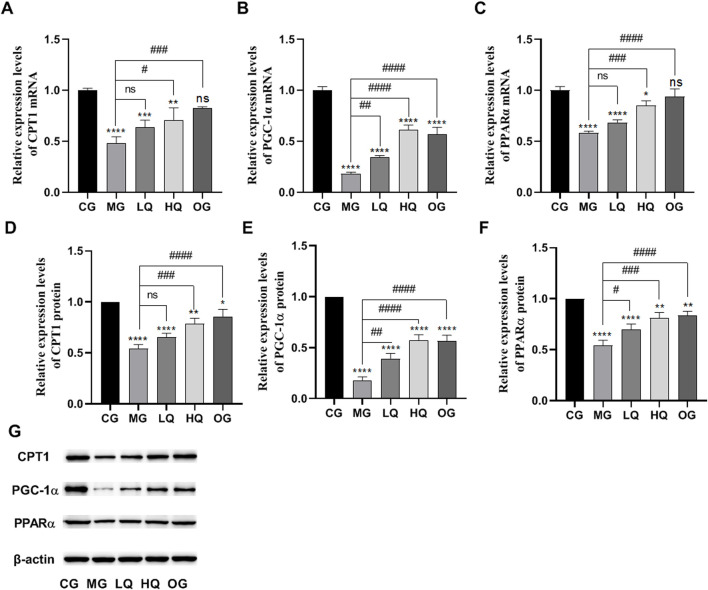
Effects of quercetin on hepatic fatty acid β-oxidation in mice with acute alcohol exposure. The mRNA expression levels of carnitine palmitoyltransferase-1 CPT1, **(A)**, peroxisome proliferator-activated receptor-γ coactivator-1α PGC-1α, **(B)**, and peroxisome proliferator-activated receptor-α PPARα, **(C)** were determined by RT-qPCR. Protein expression levels of CPT1 **(D)**, PGC-1α **(E)**, and PPARα **(F)** were analyzed by Western blotting, and the corresponding densitometric quantification is shown **(G)**. Data are expressed as mean ± SD (n = 10). *P < 0.05 and *P < 0.01 vs. CG; #P < 0.05 and ##P < 0.01 vs. MG.

As illustrated in Figure 7, hepatic mRNA and protein expression of CPT1, PGC-1α, and PPARα was markedly reduced in the MG group relative to the CG group (P < 0.01). In comparison with the MG group, administration of quercetin (HQ group) and the positive control (OG group) resulted in a significant upregulation in the expression of these genes and their corresponding proteins (P < 0.01).

## Discussion

Acute alcohol consumption is known to rapidly induce hepatic oxidative stress, inflammation, and lipid metabolic disturbances, ultimately leading to acute liver injury (Gao and Bataller, 2011; Louvet and Mathurin, 2015). In the present study, we demonstrated that quercetin pretreatment effectively attenuated acute alcohol-induced hepatic damage in mice. Quercetin improved alcohol tolerance behavior, reduced serum transaminase levels, alleviated hepatic steatosis, suppressed oxidative stress and inflammatory responses, and modulated key regulators of lipid metabolism at both transcriptional and protein levels. These findings collectively indicate that quercetin exerts hepatoprotective effects against acute alcohol-induced liver injury through coordinated regulation of lipid metabolism, oxidative stress, and inflammation.

Disruption of hepatic lipid homeostasis is a hallmark of alcohol-induced liver injury. Acute ethanol exposure promotes hepatic *de novo* lipogenesis while simultaneously impairing fatty acid β-oxidation, resulting in excessive triglyceride accumulation in hepatocytes (You and Crabb, 2004; Lieber, 2004). Previous studies have demonstrated that ethanol upregulates lipogenic enzymes such as fatty acid synthase (FAS), stearoyl-CoA desaturase-1 (SCD1), and acetyl-CoA carboxylase 1 (ACC1), while suppressing β-oxidation–related regulators including carnitine palmitoyltransferase-1 (CPT1), peroxisome proliferator-activated receptor-α (PPARα), and peroxisome proliferator-activated receptor-γ coactivator-1α (PGC-1α) (Fischer et al., 2003; Kersten, 2014). Consistent with these reports, we observed pronounced dysregulation of these pathways following acute ethanol exposure. Quercetin pretreatment markedly reversed these alterations, suppressing lipid synthesis while enhancing fatty acid oxidation, suggesting a balanced metabolic modulatory effect rather than inhibition of a single pathway.

Oxidative stress plays a central role in ethanol-induced hepatotoxicity by damaging cellular macromolecules and activating inflammatory signaling cascades (Cederbaum, 2012). Ethanol metabolism generates excessive reactive oxygen species, leading to lipid peroxidation and depletion of endogenous antioxidant defenses. In the present study, quercetin significantly reduced hepatic malondialdehyde (MDA) levels while restoring the activities of key antioxidant enzymes, including superoxide dismutase (SOD), glutathione (GSH), and catalase (CAT). These findings are consistent with previous studies reporting the potent antioxidant capacity of quercetin and its ability to enhance cellular redox homeostasis (Boots et al., 2008; Dabeek and Marra, 2019).

Inflammatory responses are closely intertwined with oxidative stress and lipid metabolic dysfunction in alcohol-induced liver injury. Acute ethanol exposure activates inflammatory signaling pathways, resulting in increased production of pro-inflammatory cytokines such as tumor necrosis factor-α (TNF-α) and interleukin-6 (IL-6), which exacerbate hepatocellular injury (Mandrekar and Szabo, 2009). In this study, quercetin pretreatment significantly suppressed the ethanol-induced elevation of these cytokines, indicating its anti-inflammatory potential. The attenuation of inflammatory responses may be attributed, at least in part, to quercetin-mediated reduction of oxidative stress and improvement of mitochondrial function, thereby limiting downstream inflammatory activation.

Quercetin has been extensively investigated in non-alcoholic fatty liver disease (NAFLD/MASLD) and chronic alcoholic liver disease models, where it has been shown to regulate lipid metabolism, oxidative stress, inflammation, and autophagy-related pathways (Li et al., 2016; Tang et al., 2012). Although autophagy-related markers were not directly assessed in the present study, the observed improvements in hepatic lipid accumulation and mitochondrial β-oxidation regulators are consistent with pathways upstream or downstream of autophagy regulation. Acute alcohol exposure has been reported to disrupt hepatic autophagy, contributing to lipid accumulation and liver injury (Ding et al., 2010). Future studies incorporating autophagy-related markers such as LC3, Beclin-1, and p62 would further clarify the involvement of this pathway in quercetin-mediated hepatoprotection.

The use of an acute alcohol-induced liver injury model represents both a strength and a limitation of this study. While chronic models better reflect long-term disease progression in humans, acute models are valuable for investigating early pathogenic events and evaluating preventive interventions against binge drinking-associated liver injury (Bertola et al., 2013). Our findings provide mechanistic insight into the early protective effects of quercetin during acute ethanol exposure; however, extrapolation of these results to chronic alcoholic liver disease or clinical settings should be approached with caution.

Several limitations of this study should be acknowledged. First, blood ethanol concentrations were not measured, which may limit precise evaluation of systemic ethanol exposure. Second, the absence of a quercetin-only control group prevents assessment of baseline hepatic effects independent of ethanol exposure. Third, renal biochemical parameters were not assessed despite histological evaluation of kidney tissues. Finally, autophagy-related signaling pathways were not directly examined. Addressing these limitations in future studies will further enhance the translational relevance of quercetin as a hepatoprotective agent.

In conclusion, this study demonstrates that quercetin effectively protects against acute alcohol-induced liver injury in mice by suppressing hepatic lipid synthesis, enhancing fatty acid β-oxidation, reducing oxidative stress, and inhibiting inflammatory responses. These findings provide new mechanistic evidence supporting the potential application of quercetin as a natural preventive strategy for alcohol-related liver injury.

## Conclusion

In conclusion, this study demonstrates that quercetin effectively protects against acute alcohol-induced liver injury in mice. Quercetin pretreatment alleviated hepatic steatosis, reduced serum transaminase levels, and attenuated oxidative stress and inflammatory responses. These protective effects are associated with the suppression of hepatic lipid synthesis and the enhancement of fatty acid β-oxidation. Collectively, our findings suggest that quercetin exerts hepatoprotective effects through coordinated regulation of lipid metabolism, oxidative stress, and inflammation, highlighting its potential as a natural preventive agent for acute alcohol-related liver injury.

## Data Availability

The original contributions presented in the study are included in the article/Supplementary Material, further inquiries can be directed to the corresponding author.
