# Supplementary material for: Quercetin attenuates acute alcohol-induced liver injury in mice by modulating lipid metabolism, oxidative stress, and inflammation
Source: Front Pharmacol. 2026 Feb 18;17:1702639. doi: 10.3389/fphar.2026.1702639 (PMC12957255; doi:10.3389/fphar.2026.1702639)
Supplement: Supplementary file 1 [file Supplementaryfile1.pdf]

Quercetin Attenuates Acute Alcohol-Induced Liver Injury in Mice by Modulating Lipid Metabolism, Oxidative Stress, and Inflammation

Figure 6G

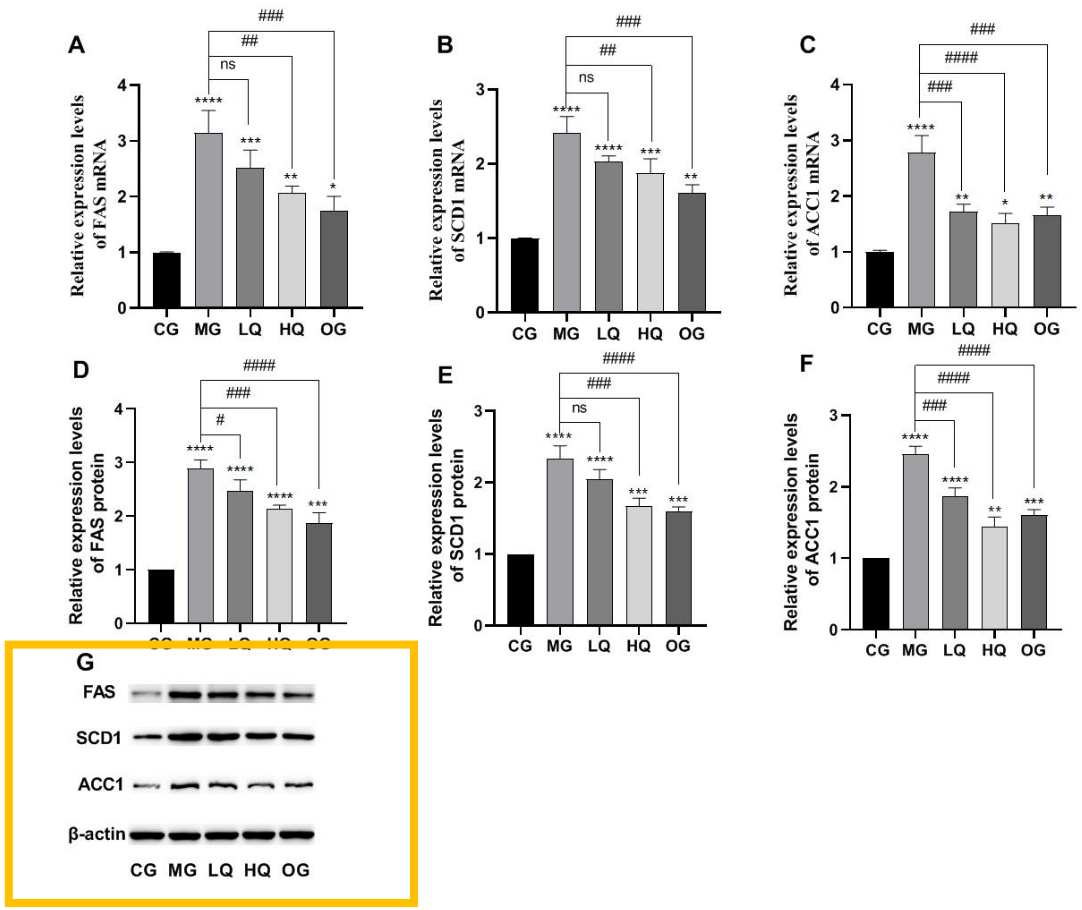
A
Relative expression levels of FAS mRNA
B
Relative expression levels of SCD1 mRNA
C
Relative expression levels of ACC1 mRNA
D
Relative expression levels of FAS protein
E
Relative expression levels of SCD1 protein
F
Relative expression levels of ACC1 protein
G
FAS
SCD1
ACC1
β-actin
CG MG LQ HQ OG

FAS

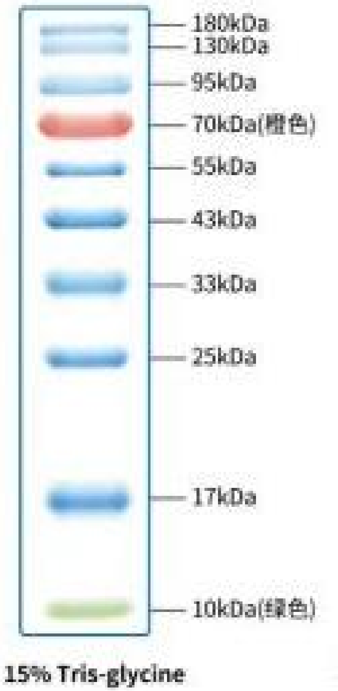
180kDa
130kDa
95kDa
70kDa(橙色)
55kDa
43kDa
33kDa
25kDa
17kDa
10kDa(绿色)
15% Tris-glycine

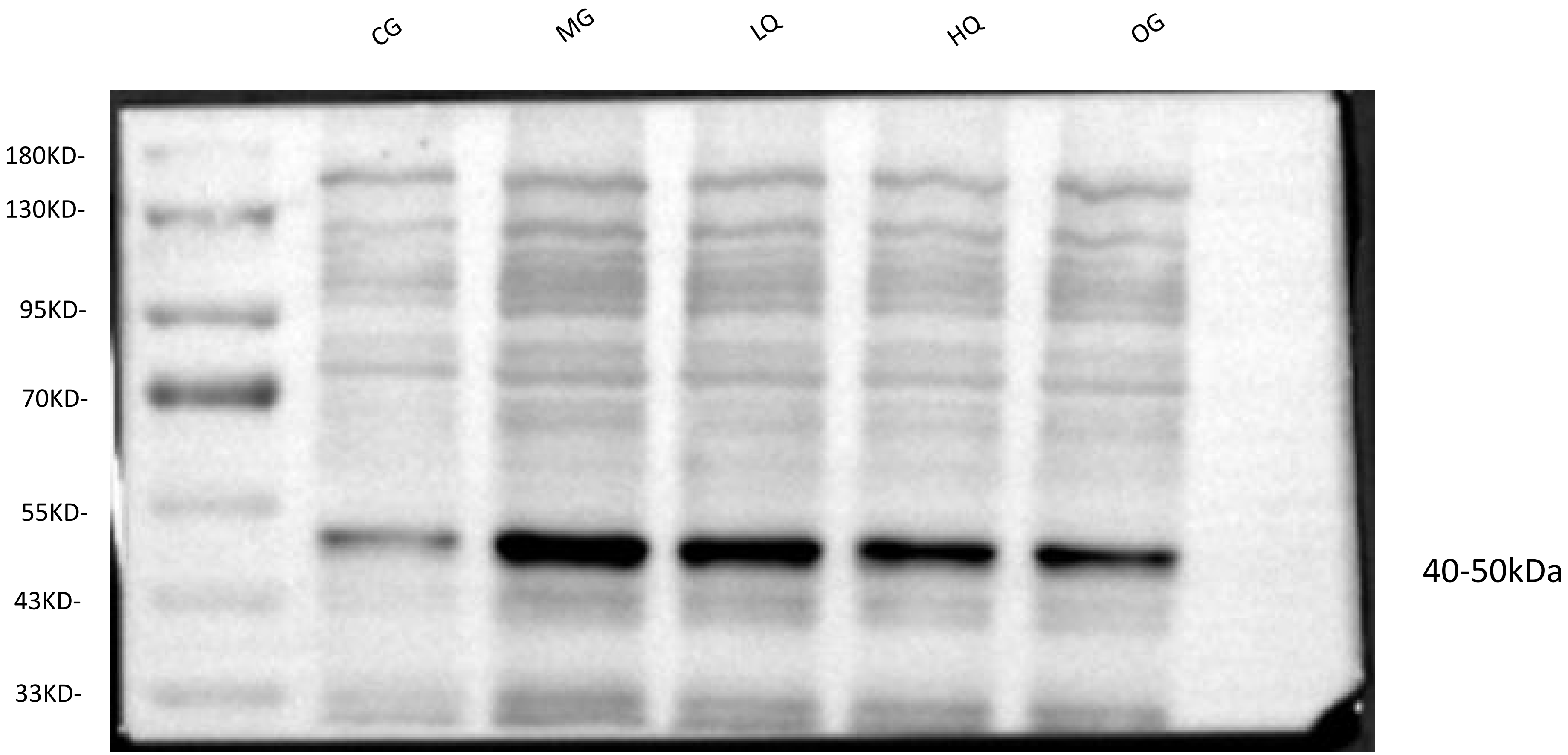
CG
MG
LQ
HQ
OG
180KD-
130KD-
95KD-
70KD-
55KD-
43KD-
33KD-
40-50kDa

SCD1

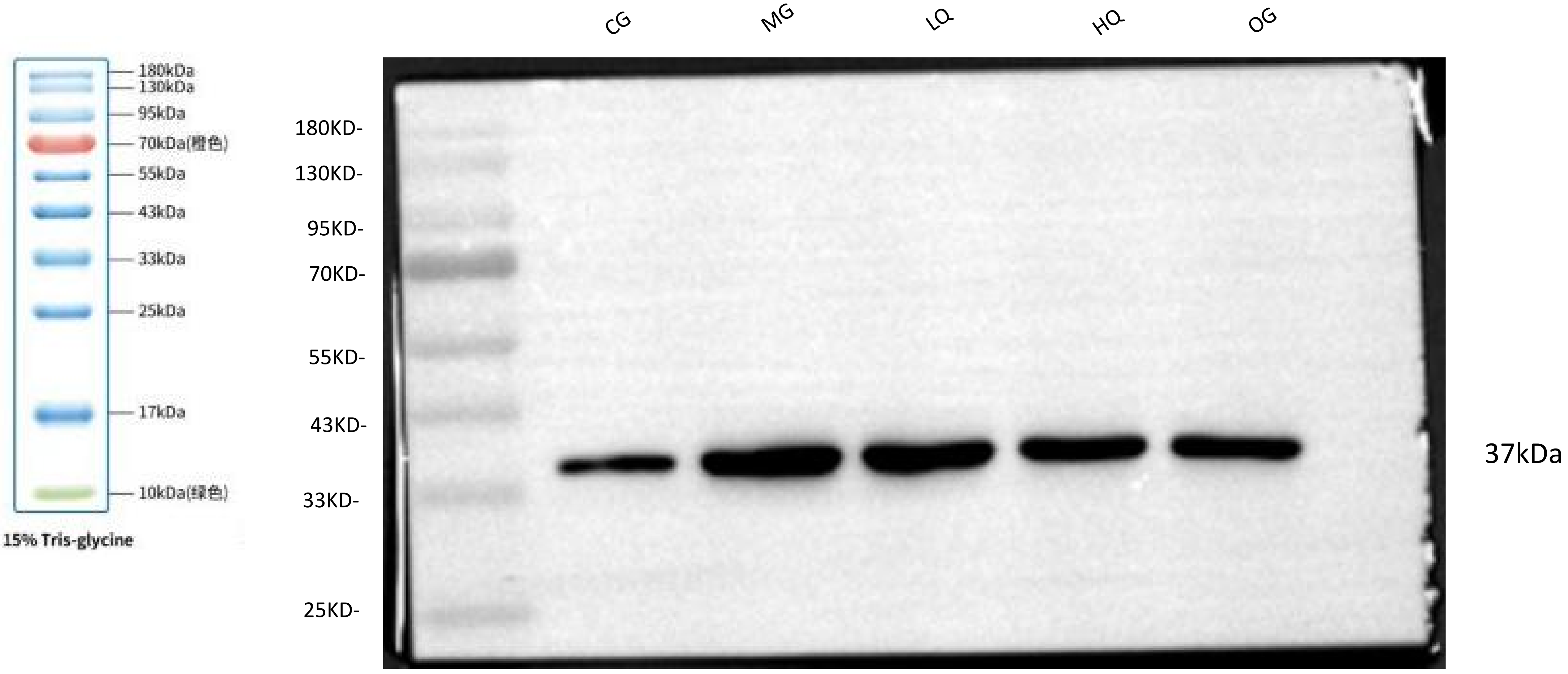
180kDa
130kDa
95kDa
70kDa(橙色)
55kDa
43kDa
33kDa
25kDa
17kDa
10kDa(绿色)
15% Tris-glycine
CG
MG
LQ
HQ
OG
180KD-
130KD-
95KD-
70KD-
55KD-
43KD-
33KD-
25KD-
37kDa

ACC1

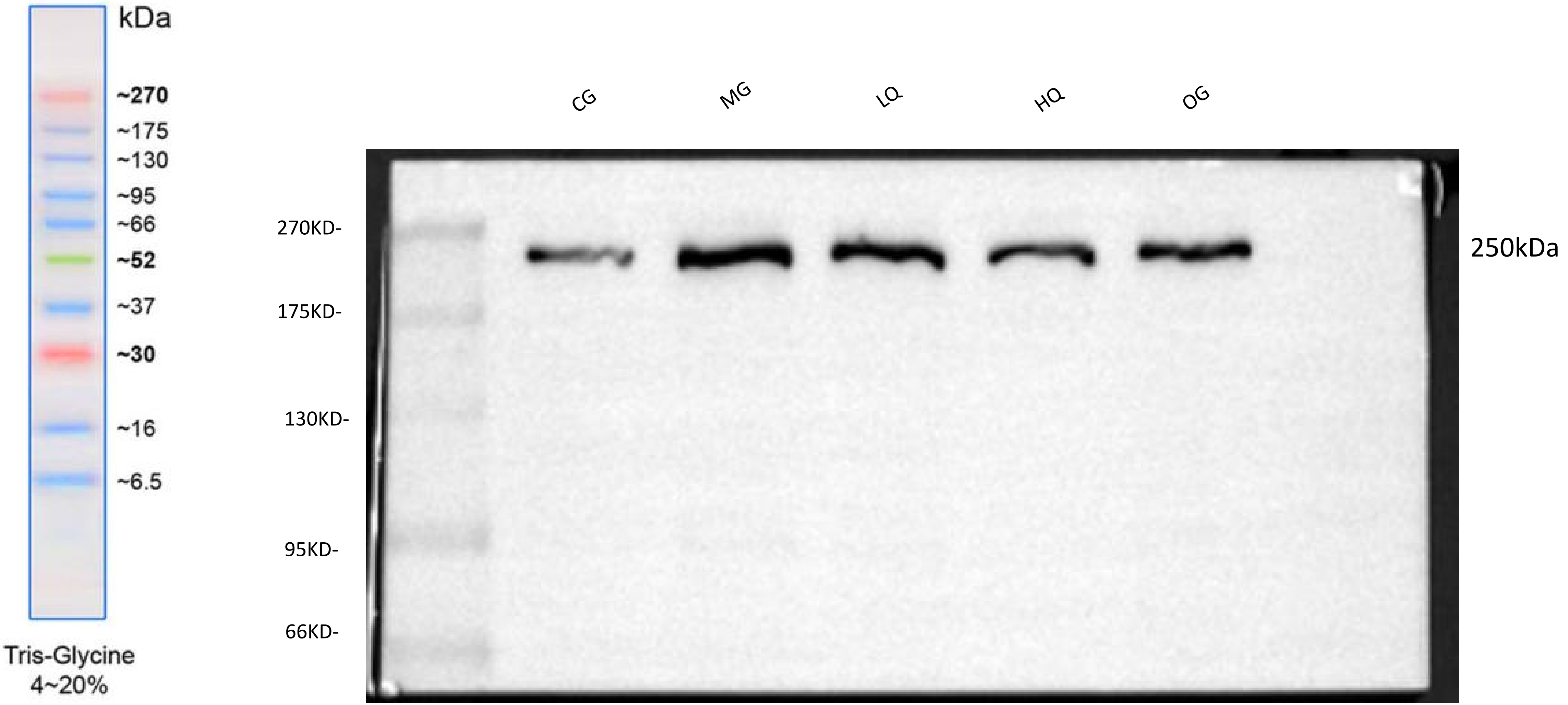
kDa
~270
~175
~130
~95
~66
~52
~37
~30
~16
~6.5
Tris-Glycine
4~20%
CG
MG
LQ
HQ
OG
270KD-
175KD-
130KD-
95KD-
66KD-
250kDa

β-actin

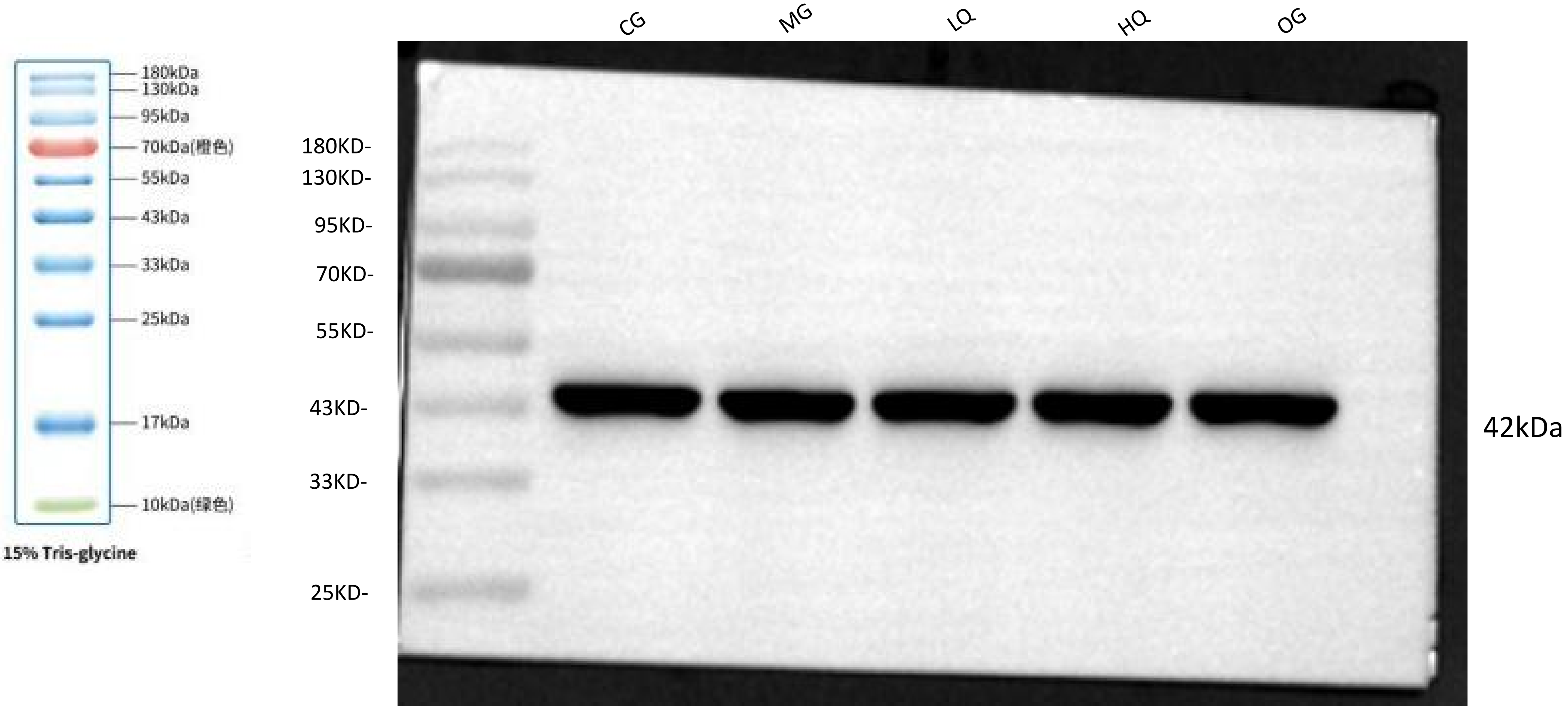

180kDa
130kDa
95kDa
70kDa(橙色)
55kDa
43kDa
33kDa
25kDa
17kDa
10kDa(绿色)
15% Tris-glycine
CG
MG
LQ
HQ
OG
180KD-
130KD-
95KD-
70KD-
55KD-
43KD-
33KD-
25KD-
42kDa

Figure 7G

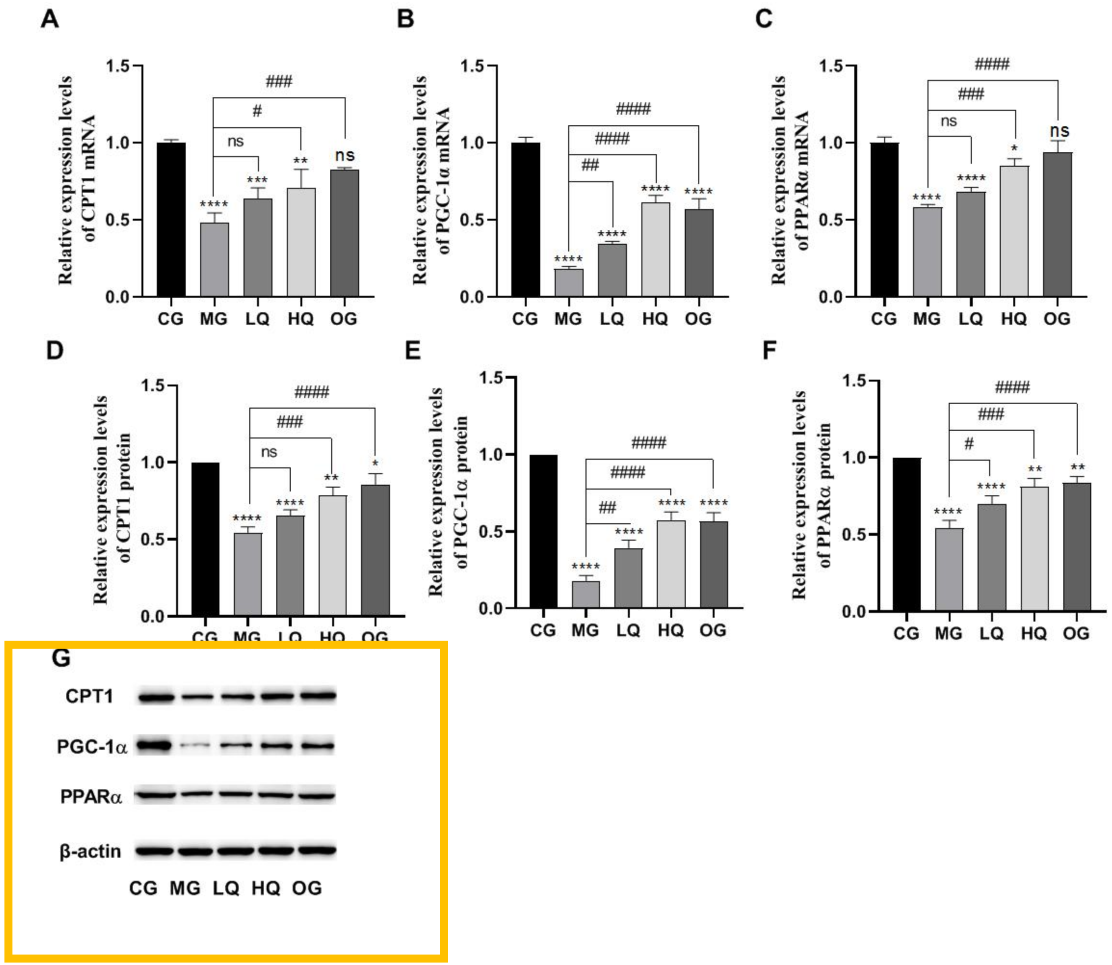

A
B
C
D
E
F
G
Relative expression levels of CPT1 mRNA
Relative expression levels of PGC-1α mRNA
Relative expression levels of PPARα mRNA
Relative expression levels of CPT1 protein
Relative expression levels of PGC-1α protein
Relative expression levels of PPARα protein
CG MG LQ HQ OG
CPT1
PGC-1α
PPARα
β-actin

CPT1

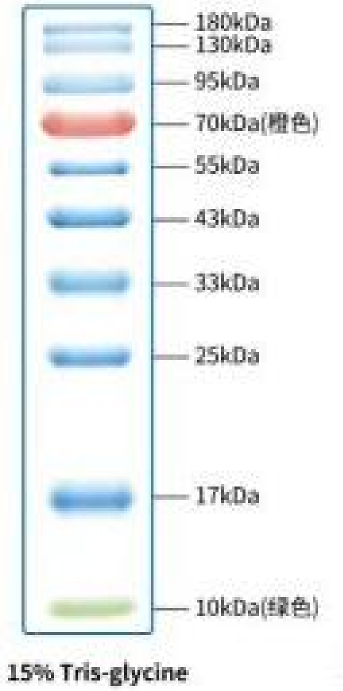
180kDa
130kDa
95kDa
70kDa(橙色)
55kDa
43kDa
33kDa
25kDa
17kDa
10kDa(绿色)
15% Tris-glycine

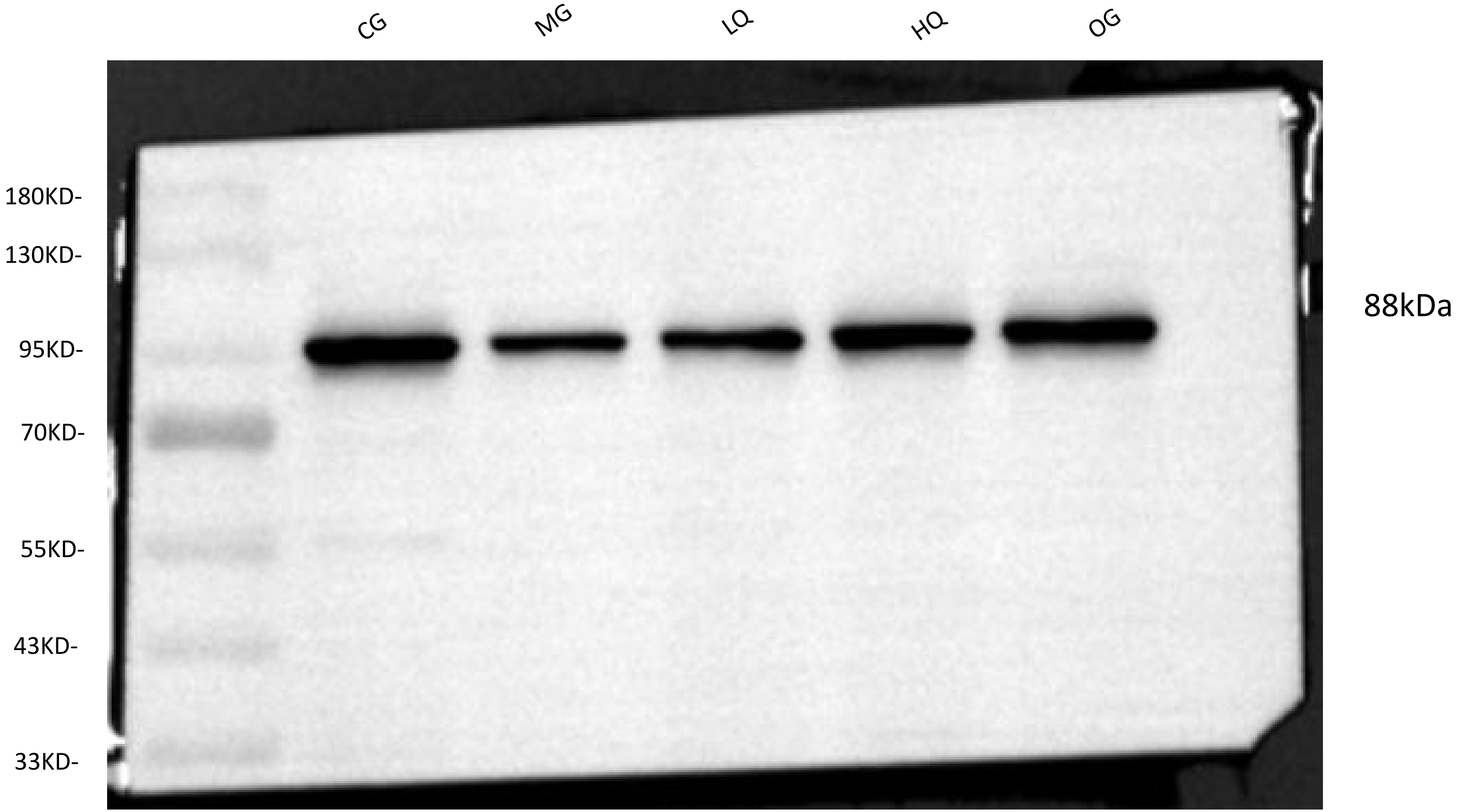
CG
MG
LQ
HQ
OG
180KD-
130KD-
95KD-
70KD-
55KD-
43KD-
33KD-
88kDa

PGC-1α

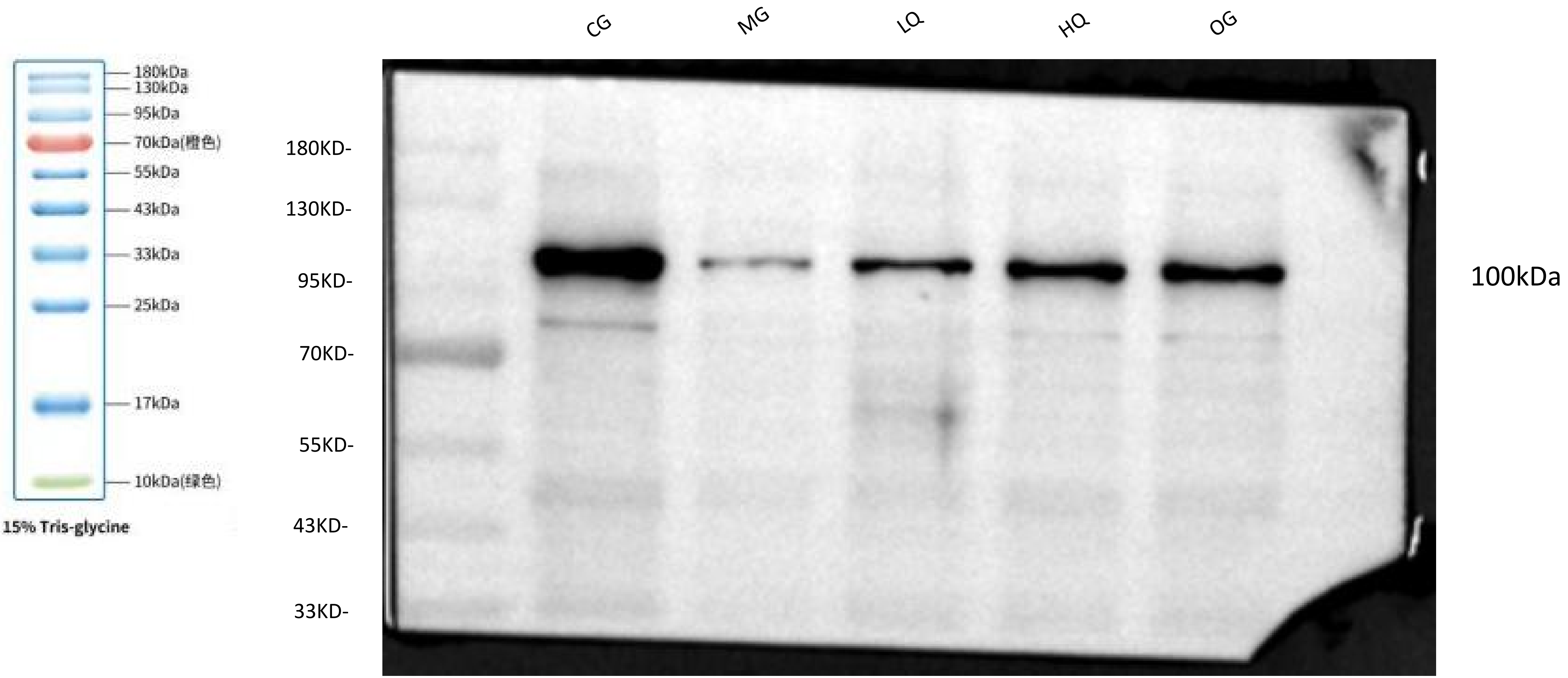

180kDa
130kDa
95kDa
70kDa(橙色)
55kDa
43kDa
33kDa
25kDa
17kDa
10kDa(绿色)
15% Tris-glycine
CG
MG
LQ
HQ
OG
180KD-
130KD-
95KD-
70KD-
55KD-
43KD-
33KD-
100kDa

PPARα

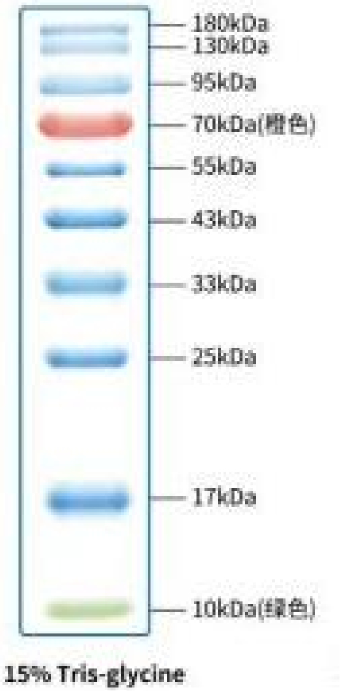
180kDa
130kDa
95kDa
70kDa(橙色)
55kDa
43kDa
33kDa
25kDa
17kDa
10kDa(绿色)
15% Tris-glycine

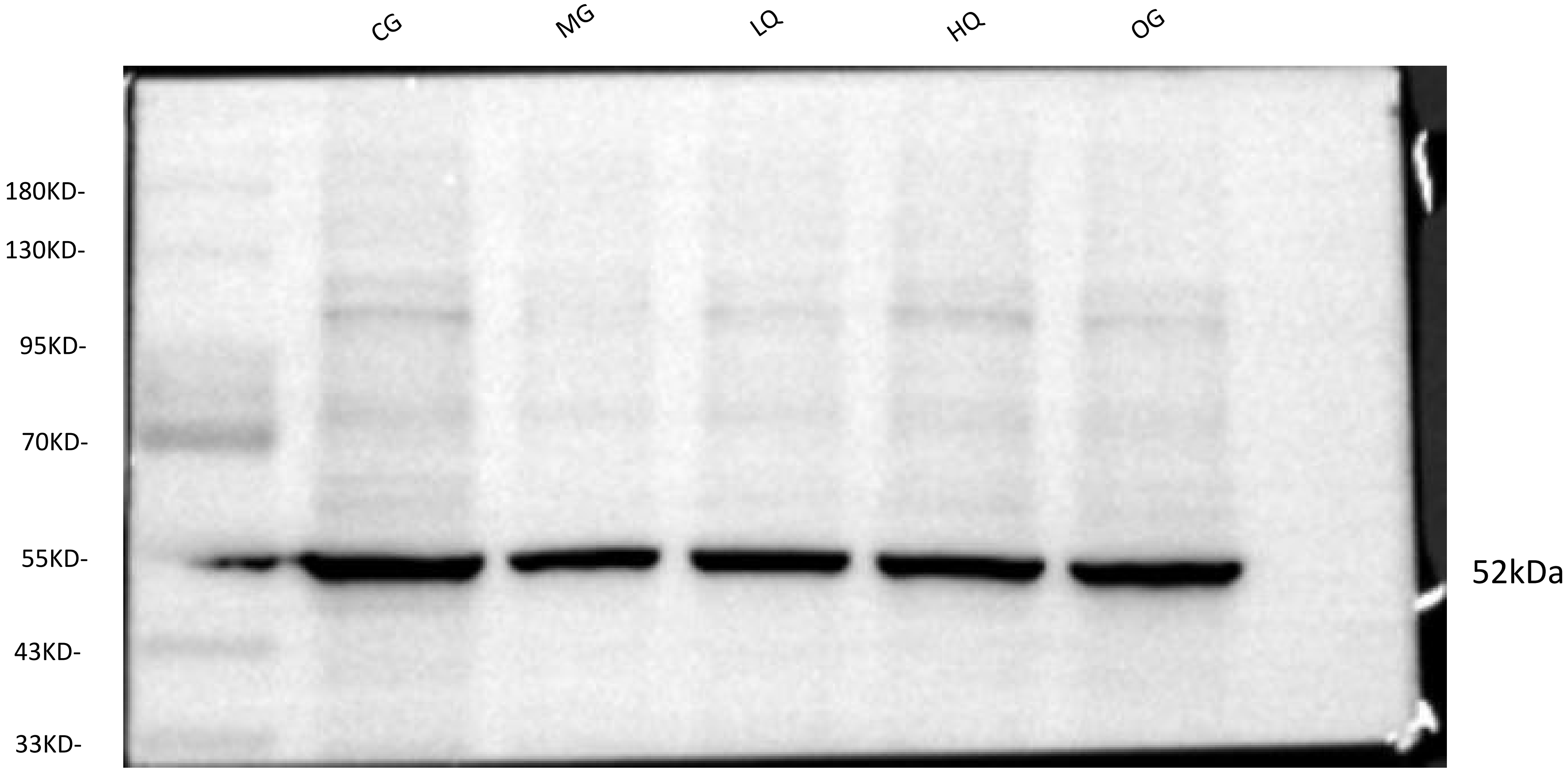
CG
MG
LQ
HQ
OG
180KD-
130KD-
95KD-
70KD-
55KD-
43KD-
33KD-
52kDa

β-actin

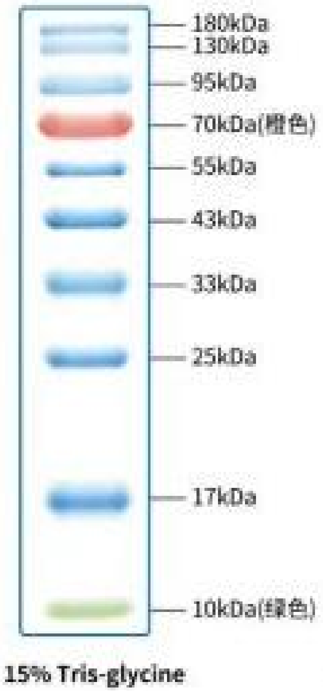

180kDa
130kDa
95kDa
70kDa(橙色)
55kDa
43kDa
33kDa
25kDa
17kDa
10kDa(绿色)
15% Tris-glycine

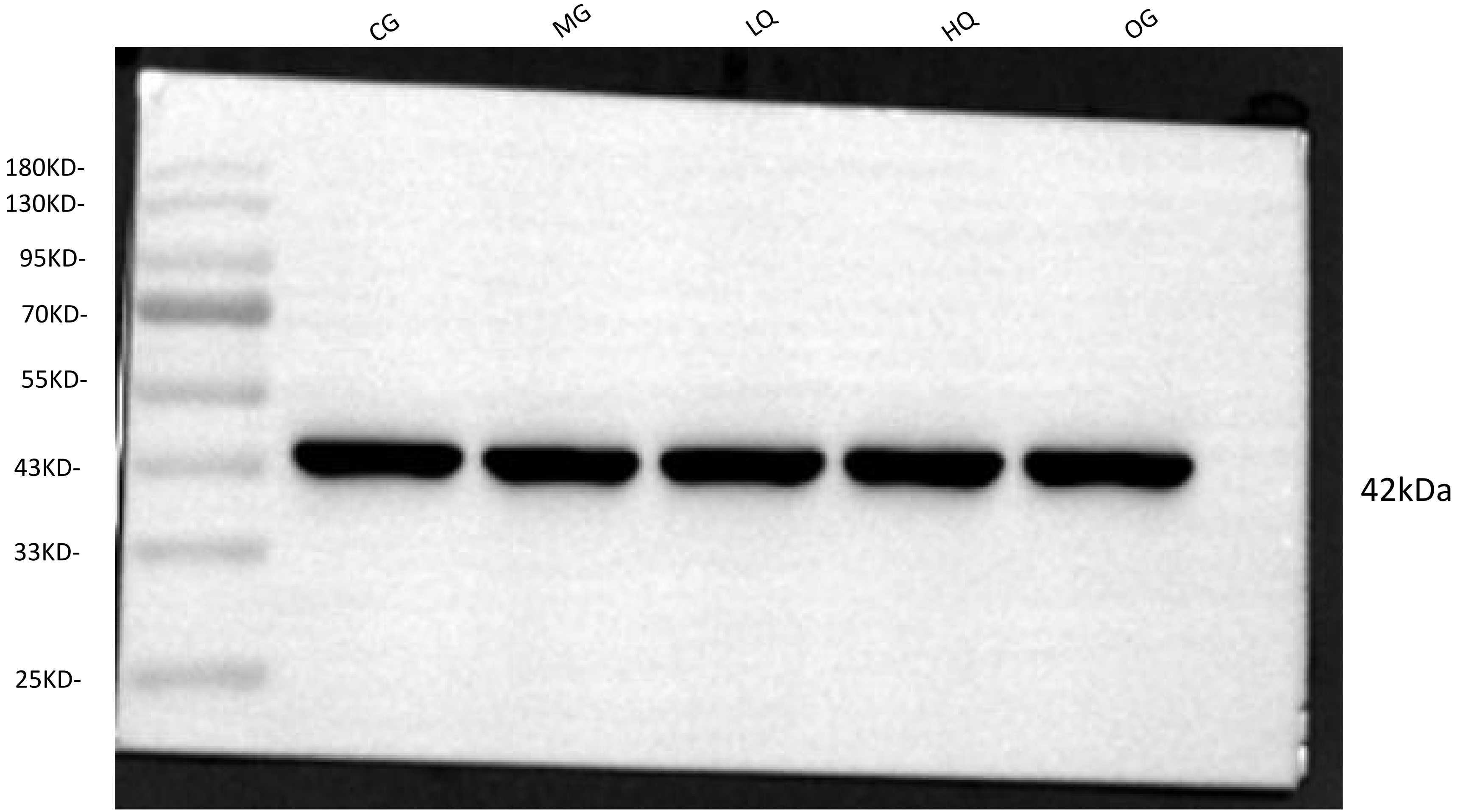

CG
MG
LQ
HQ
OG
180KD-
130KD-
95KD-
70KD-
55KD-
43KD-
33KD-
25KD-
42kDa
